# A functional variant of *SHARPIN* confers increased risk of late-onset Alzheimer’s disease

**DOI:** 10.1038/s10038-021-00987-x

**Published:** 2021-11-05

**Authors:** Yuya Asanomi, Daichi Shigemizu, Shintaro Akiyama, Akinori Miyashita, Risa Mitsumori, Norikazu Hara, Takeshi Ikeuchi, Shumpei Niida, Kouichi Ozaki

**Affiliations:** 1 Medical Genome Center, Research Institute, National Center for Geriatrics and Gerontology, Obu, Aichi Japan; 2 Department of Medical Science Mathematics, Medical Research Institute, Tokyo Medical and Dental University, Tokyo, Japan; 3 RIKEN Center for Integrative Medical Sciences, Yokohama, Japan; 4 Department of Molecular Genetics, Brain Research Institute, Niigata University, Niigata, Japan

**Keywords:** Genetic association study, Alzheimer's disease

## Abstract

Late-onset Alzheimer’s disease (LOAD) is the most common form of dementia, and its pathogenesis is multifactorial. We previously reported a rare functional variant of *SHARPIN* (rs572750141, NP_112236.3:p.Gly186Arg) that was significantly associated with LOAD. In addition, several recent studies have suggested the potential role of SHARPIN in AD pathogenesis. In this study, we sought to identify additional functional variants of *SHARPIN* in Japanese population. Six highly deleterious variants of *SHARPIN*, comprising four missense variants, one frameshift variant, and one stop-gain variant were detected from whole-genome sequencing data for 180 patients with LOAD and 184 with mild cognitive impairment. One of these candidate variants (rs77359862, NP_112236.3:p.Arg274Trp) was significantly associated with an increased risk of LOAD in 5043 LOAD cases and 11984 controls (*P* = 0.0016, odds ratio = 1.43). Furthermore, this variant SHARPIN showed aberrant cellular localization and reduced the activation of NF-κB, a central mediator of inflammatory and immune responses. Further investigation of the physiologic role of SHARPIN may reveal the mechanism of onset of LOAD.

## Introduction

Dementia is associated with a deterioration in cognitive function and is one of the leading causes of death worldwide. The number of elderly adults with dementia is rapidly increasing and expected to reach 74.7 million in 2030 and 131.5 million in 2050 [1]. Alzheimer’s disease (AD), comprising familial (early-onset), and sporadic (late-onset) disease forms, is the most common form of dementia [2]. Currently, the only available treatment for late-onset AD (LOAD) is to decelerate the progression of the disease.

LOAD arises from complex interactions among multiple genetic and environmental factors [3, 4]. A large twin study in 2006 revealed an estimated heritability (*h*^2^) of 58–79% for LOAD [5]. Since the 2010s, meta-analysis of genome-wide association studies (GWAS) has identified more than 40 loci associated with the risk of LOAD [6–8]. The *APOE* ε4 allele is the strongest known genetic risk factor for LOAD. However, combining all known risk loci fails to account for the total estimated heritability of LOAD. To breach this missing heritability, next-generation sequencing technologies such as whole-genome sequencing (WGS) and whole-exome sequencing (WES) have been applied to discover LOAD-risk variants. Although these studies based on Caucasian cohorts have revealed functional variants in several genes, including *TREM2* [9–11], the identified variants have rarely occurred in Japanese populations.

The multifunctional SHARPIN (SHANK-associated RH domain interactor) protein is associated with numerous physiologic functions and many diseases. Initially, SHARPIN was found as a post-synaptic density protein [12]. A well-known function of SHARPIN is its participation in formation of the linear ubiquitination assembly complex (LUBAC), which regulates the NF-κB activation pathway, a central mediator of inflammatory and immune responses [13–16]. Recently, we conducted WES of 202 Japanese LOAD patients without the *APOE* ε4 risk allele [17]. *SHARPIN* was found as one of ten significant genes on a gene-based analysis in the process of the candidate variants discovery. Finally, we found a rare functional variant of *SHARPIN* (rs572750141, NP_112236.3:p.Gly186Arg) that is associated with an increased risk of LOAD (odds ratio = 6.1). Subsequent functional analyses revealed that this variant SHARPIN protein (G186R) resulted in aberrant cellular localization and attenuated the activation of NF-κB [17]. In addition, during the past decade, various LUBAC formation-dependent and -independent functions of SHARPIN have been revealed in association with, for example, many types of cancers [18–25], tumor necrosis factor-α (TNF-α)–induced cell death [26], and regulation of caspase 1 activity in sepsis [27]. Most notably, SHARPIN is suggested to play a role in AD pathogenesis [28, 29]. Amyloid-β-induced oxidative stress enhances SHARPIN expression in macrophages, and SHARPIN regulates amyloid-β phagocytosis and the expression of NLRP3, which forms inflammasomes and is activated in AD. Therefore, further exploration of genetic variants in *SHARPIN* may lead to the discovery of additional variants with noteworthy effects on LOAD risk.

Here, we report a novel variant of *SHARPIN* (rs77359862, NP_112236.3:p.Arg274Trp) that is associated with an increased risk of LOAD. We identified this variant through in silico analysis of WGS data and a large-scale association study involving Japanese population. This variant SHARPIN shows altered intracellular localization and decreased TNF-α-induced activation of NF-κB.

## Materials and methods

### Study population

For WGS, we used genomic DNA samples from 180 patients with LOAD and 184 with mild cognitive impairment (MCI) that were registered with the National Center for Geriatrics and Gerontology (NCGG) Biobank. These patients were diagnosed at the NCGG Hospital according to the criteria of the National Institute on Aging and the Alzheimer’s Association [30, 31]. The first cohort of the association study consisted of 1763 patients with LOAD and 3214 controls who were recruited from the NCGG Biobank; the second cohort comprised 3280 LOAD cases and 8770 controls (2321 LOAD cases and 2636 controls from Niigata University; 688 LOAD cases from the BioBank Japan Project [32, 33]; 916 controls from the Pharma SNP Consortium; 425 controls from the Japan Biological Informatics Consortium; and 271 AD cases and 4793 controls from the NCGG Biobank, independent of 1st cohort). All subjects were of Japanese origin and provided written informed consent. The study was performed with the approval of the ethics committee of each institution.

### WGS data analysis

Library preparation by using a TruSeq DNA PCR-Free Library Preparation Kit (Illumina, San Diego, CA) and sequencing by using the Illumina HiSeq X Ten or NovaSeq 6000 platform (2 × 151 bp paired-end reads) were conducted at Macrogen Japan (Tokyo, Japan), Takara Bio (Shiga, Japan), and GENEWIZ (South Plainfield, NJ, USA). FASTQ-formatted read sequences were mapped to the reference human genome (hg19) by using Burrows–Wheeler Alignment–MEM (version 0.7.15) [34]. Duplicate reads were removed by applying Picard (version 2.21.4) [35]. Variant calling was performed by using the Genome Analysis Toolkit (GATK; version 4.1.0.0) according to GATK Best Practice recommendations [36, 37]. All variants were annotated by using snpEff (version 4.3) [38] and ANNOVAR (version 20180416) [39].

### In silico association study

By using the GAS (Genetic Association Study) Power Calculator (http://csg.sph.umich.edu/abecasis/cats/gas_power_calculator/index.html), we first calculated the study power (1 − β) for each minor allele frequency (MAF) of six candidate variants and the study sample size. We then applied the corrected significance level (*α* = 0.05/6 = 0.0083), prevalence (0.1), and relative risk (6.1). Relative risk was set according to the odds ratio for rs572750141, as shown in our previous study [17]. Genotyping data were downloaded from the NCGG Biobank database. All 17,027 subjects from both the first and second cohorts were genotyped by using the Infinium Asian Screening Array (Illumina) according to the manufacturer’s protocol. The genotype of the rs77359862 allele was extracted from the VCF-formatted data by using PLINK software (version 1.9) [40]. Statistical analyses were performed by using R software (version 3.6.0). *P* values were calculated by using *χ*^2^ test. Odds ratios and 95% confidence intervals were calculated by using the vcd package (version 1.4.4) in R. Meta-analyses were performed by using the Mantel–Haenszel *χ*^2^ test with continuity correction.

### Genotyping

We obtained the genomic DNA of 1763 LOAD cases and 3214 controls from the NCGG Biobank, which consistent individuals with the 1st cohort. We genotyped a candidate variant, rs1378764618, by using a multiplex PCR Invader assay (Third Wave Technologies, Madison, WI, USA) [41] and QuantStudio 7 Flex Real-Time PCR System (Thermo Fisher Scientific, Waltham, MA, USA).

### Primers and construction of plasmids

Primers for PCR reactions were designed by using the Primer3Plus program (http://primer3plus.com/cgi-bin/dev/primer3plus.cgi) and were synthesized commercially (Fasmac, Kanagawa, Japan). Plasmids for Myc-SHARPIN (wild-type and G186R) were based on the pCMV-Myc vector and were constructed previously [17]. Site-directed mutagenesis for the construction of the plasmid with R274W variant was performed by using PrimeSTAR Max DNA Polymerase (Takara Bio); the primer set for mutagenesis (5′-CATCGGATGGTGCCTGTGTGTGCCTG-3′ and 5′-AGGCACCATCCGATGACCCAGCGTTG-3′; the mutated site is underlined) was designed according to the manufacturer’s instructions. The PCR mix for mutagenesis contained 1× PrimeSTAR Max Premix, 0.2 μM of each primer, and 70 pg wild-type Myc-SHARPIN plasmid in a total reaction volume of 50 μl. The cycling conditions were: 30 cycles of 98 °C for 10 s, 55 °C for 15 s, and 72 °C for 25 s. The PCR product was used to transform *Escherichia coli* strain DH5α cells and the inserted sequence was confirmed via Sanger sequencing.

### Sanger sequencing

For validation of the variants found by using WGS data, purified PCR products underwent Sanger sequencing by using *Taq* DNA Polymerase (Genscript, Piscataway, NJ, USA), a BigDye Terminator v3.1 Cycle Sequencing Kit, and an ABI 3100 or 3500 Genetic Analyzer (Thermo Fisher Scientific).

### Luciferase assay

We used a previously constructed stable HEK293 cell line containing the luciferase reporter plasmid pGL4.32[luc2P/NF-κB-RE/Hygro] (Promega, Madison, WI, USA) [17]. Cells were plated in 96-well plates (1.5 × 10^4^ cells/well) and were cultured in Dulbecco’s Modified Eagle Medium (DMEM) for 24 h before transfection with the plasmid and FuGENE HD Transfection Reagent (Promega). Transfected cells were cultured for 24 h and then treated with 20 ng/ml TNF-α (Wako, Osaka, Japan) for 5 h. The Nano-Glo Dual-Luciferase Reporter Assay System (Promega) was used to measure luciferase activity. We performed three independent experiments with five replicate samples each; Student’s *t* test was used for statistical analysis of these results.

### Immunocytochemistry

HEK293 cells (2.0 × 10^4^ cells/well) were plated on BioCoat Poly-D-Lysine 4-well Culture Slides (Corning, NY, USA) and cultured in DMEM for 24 h. Then cells were transfected with the Myc-SHARPIN plasmids by using FuGENE HD Transfection Reagent (Promega), fixed for 24 h after transfection, and then incubated with Anti-Myc-tag mAb-Alexa Fluor 488 (MBL, Nagoya, Japan) according to the manufacturer’s protocol. The slides were mounted by using SlowFade Diamond Antifade Mountant with DAPI (Thermo Fisher Scientific). Fluorescence images were obtained on a BIOREVO BZ-9000 fluorescence microscope (Keyence, Osaka, Japan).

## Results

### *SHARPIN* coding variants in Japanese WGS data

To find novel risk variants of *SHARPIN* among Japanese population, we obtained the genotypes for the coding region of 180 patients with LOAD and 184 with MCI from WGS data (Table 1).

**Table 1:** Demographic features of patients with LOAD or MCI in WGS data

	LOAD	MCI	Total
Number of patients	180	184	364
Male/female	70/110	96/88	166/198
Mean age (years; 1 standard deviation)	67.5 (9.8)	69.4 (9.3)	68.4 (9.7)

**Fig. 1:**
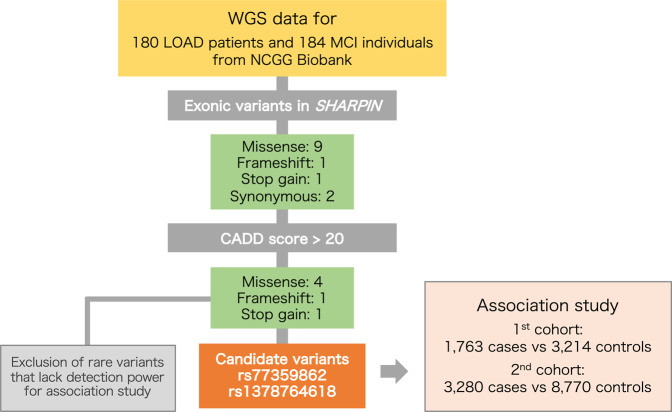
Overview of the LOAD-risk variant discovery workflow

Before the risk variant examination (Fig. 1), we confirmed the absence of known mutations in causal genes—*APP*, *PSEN1*, and *PSEN2*—for autosomal-dominant early-onset AD. In addition, the rare risk variant previously we found, rs572750141, was not found. We then extracted 13 exonic variants of *SHARPIN* (nine missense variants, one frameshift variant, one stop-gain variant, and two synonymous variants; Table S1) from the WGS data. We annotated these 13 variants according to the Combined Annotation Dependent Depletion score [42], which indicates the deleteriousness of variants in the human genome. This process returned six potentially highly deleterious (scaled C score, >20) variants: four missense variants, one frameshift variant, and one stop-gain variant (Table 2). These six variants identified by analyzing WGS data were validated by performing Sanger sequencing of genomic DNA from the corresponding subjects.

**Table 2:** Possible functional exonic variants of *SHARPIN* (CADD > 20) in WGS data

Position (hg19)	Ref/Alt	dbSNP	Population frequency in public database			Protein	CADD score	No. of carriers	
			gnomAD (Total)	gnomAD (East Asian)	8.3KJPN			LOAD	MCI
8:145153803	G/C	NA	NA	NA	NA	p.Pro381Arg	24.3	0	1
8:145153808	C/T	NA	NA	NA	NA	p.Trp379^*^	37.0	0	1
8:145153873	T/C	rs201818510	0.000013	0.00018	0.0001	p.Thr358Ala	24.8	0	1
8:145154035	C/–	NA	NA	NA	NA	p.Leu333fs	21.4	1	0
8:145154230	T/C	rs1378764618	0.0000040	0.000056	0.0029	p.Asp291Gly	26.7	0	2
8:145154282	G/A	rs77359862	0.0030	0.038	0.011	p.Arg274Trp	25.8	1	6

*NA* Not available

### Association study

We then assessed the association between each of these six highly deleterious variants and LOAD through a two-stage process involving the genotypes of 1763 cases and 3214 controls for the first stage and 3280 cases and 8770 controls for the replication stage (Table S2). However, four of the variants had very low MAF: three were novel singleton variants, and the MAF of rs201818510 was <0.02% in both the East Asian (gnomAD: Genome Aggregation Database) and Japanese (8.3KJPN) genomic databases (Table 2). The association analysis for these variants with low MAF had insufficient statistical power (1 − β < 0.4) in the sample size of our population. In contrast, the power calculated for rs1378764618 and rs77359862 (MAF = 0.0029 and 0.011, respectively, in 8.3KJPN) was higher (i.e., 1 − β = 1.0). Therefore, we conducted association analyses for rs1378764618 and rs77359862 with LOAD in the Japanese population.

This analysis disclosed a significant association of rs77359862 with LOAD (Bonferroni-corrected *P* = 0.024); rs1378764618 lacked a significant association with LOAD (Table S3). The association between rs77359862 and LOAD was validated (*P* = 0.029) (Table 3) with the second cohort (3280 LOAD cases and 8770 controls). Finally, meta-analysis showed a significant association between rs77359862 and LOAD (*P* = 0.0016) and identified rs77359862 as a novel *SHARPIN* variant that confers an increased risk of LOAD (odds ratio = 1.43) (Table 3).

**Table 3:** Summary of association study of rs77359862 with the risk of LOAD

Phase	No. of samples		No. of variants Hetero (Homo)		MAF		Odds ratio	95% CI	*P*
	Cases	Controls	Cases	Controls	Cases	Controls			
1st cohort	1763	3214	47 (1)	55	0.014	0.0086	1.63	1.11–2.41	0.012
2nd cohort	3280	8770	83	166	0.013	0.0095	1.34	1.03–1.75	0.029
Combined^a^	5043	11984	130 (1)	221	0.013	0.0092	1.43	1.15–1.78	0.0016

*MAF* minor allele frequency, *CI* confidence interval

^a^*P* value was calculated by using Mantel–Haenszel test

### Functional analysis of R274W SHARPIN variant

**Fig. 2:**
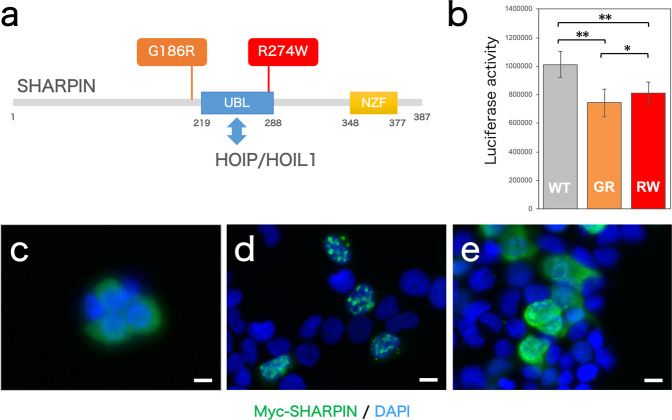
Effect of the R274W variant on SHARPIN function. **a** This schematic diagram shows the domain architecture of the SHARPIN protein and the positions of the variants on which our current and previous studies were focused. UBL, ubiquitin-like domain; NZF, Nlp4 zinc finger domain. **b** NF-κB activity in HEK293 cells under TNF-α–induced activation was determined via luciferase assay, which was performed three times with five replicates in each assay. WT wild-type, GR G186R, RW R274W. *P < 0.05; **P < 0.005. **c**–**e** Localization in HEK293 cells of Myc-SHARPIN (**c**) wild-type, (**d**) G186R, and (**e**) R274W was visualized via immunocytochemistry. Scale bar, 10 µm

The identified LOAD-risk variant, rs77359862, results in an amino acid change in SHARPIN (p.Arg274Trp; R274W). The arginine residue at position 274 of SHARPIN is located in the ubiquitin-like domain (Fig. 2a), which interacts with HOIP, a catalytically active component of LUBAC, while previously reported LOAD-risk variant G186R lies near the ubiquitin-like domain. We therefor analyzed the functional effects of the R274W SHARPIN variant.

First, we used a luciferase assay to evaluate the effect of the R274W variant on NF-κB activity (Fig. 2b). We found significantly decreased NF-κB activity in HEK293 cells when the R274W-type Myc-SHARPIN was expressed compared with the wild-type Myc-SHARPIN; the G186R-type Myc-SHARPIN likewise significantly decreased NF-κB activity, thus supporting our previous findings [17]. We then used immunocytochemistry to examine the cellular localization of SHARPIN protein variants (Figs. 2c–e, S1). In HEK293 cells, wild-type Myc-SHARPIN was uniformly distributed throughout the cytosol. In contrast, G186R-type Myc-SHARPIN was present as uneven clumps of granules, as we noted previously [17]; the R274W-type Myc-SHARPIN also produced cytoplasmic clumping.

## Discussion

Here, we identified six candidate LOAD-risk variants of *SHARPIN* by analyzing the WGS data of 180 Japanese patients with LOAD and 184 with MCI (Table 2). One of these six candidates, a novel functional coding variant of *SHARPIN* (rs77359862, NP_112236.3:p.Arg274Trp), was significantly associated with an increased risk of LOAD (Table 3). Furthermore, functional analysis in cells revealed that the R274W variant altered the localization of the SHARPIN protein and reduced the activation of NF-κB, which is located downstream of SHARPIN in the signaling pathway (Fig. 2). Because of their rarity, the statistical association of four of the six candidate variants with LOAD could not be assessed owing to a lack of power (1 − β < 0.4) depends on the insufficient sample size in this study. However, these variants, which include both nonsense and frameshift mutations, might also demonstrate aberrant SHARPIN function. Therefore, determining the association between these additional variants and LOAD in a large Japanese cohort is warranted.

Compared with the rare SHARPIN variant we previously reported (rs572750141, odds ratio = 6.1) [17], the variant we found in the current study (rs77359862) carries a relatively modest risk of LOAD (odds ratio = 1.43), consistent with its milder phenotype in the functional analysis. These results indicated that, compared with the G186R mutant, the R274W variant causes less aberrant localization of SHARPIN with less reduction in NF-κB activity and thus confers milder risk of the onset of LOAD. However, the odds ratio of rs77359862 is higher than that for many GWAS SNPs of LOAD, except the *APOE* ε4 allele (rs429358). In addition, the proportion of carriers of rs77359862 is 1 to 4% in Japanese (or East Asian) cohorts, which is more frequent than for the rs572750141 (<0.05% in Japanese), thus suggesting the potential clinical importance of rs77359862.

Recent studies on the pathogenic mechanism of LOAD have focused on the immune function of the nervous system, such as the important role of microglia [9, 10]. For example, a variant of *TREM2*, previously reported as a LOAD-risk factor in Caucasian cohort studies, affects Aβ phagocytosis by microglia [43]. The functional variant of *SHARPIN* that we identified here might also increase the risk of LOAD onset by altering nervous system immune function. In addition to the effects of SHARPIN on the NF-κB pathway, as we studied here, SHARPIN exerts various functions [12–16, 18–29]. For example, by modulating linear ubiquitination, LUBAC induces proteasomal degradation of aberrantly aggregated proteins, including mutant Huntingtin, Ataxin-3, SOD1, and TDP-43, which all are involved in neurodegenerative disease [44]. Furthermore, the immunoreactivity of the linear polyubiquitin chain was identified in tau pathology of LOAD [45]. Therefore, investigating the influence of SHARPIN variants on these broad functions may provide insight into the mechanism underlying the onset of LOAD.

In addition to our previous report on rs572750141 [17], a recent study by the ADNI (Alzheimer’s Disease Neuroimaging Initiative) reported a significant association of the *SHARPIN* coding variant rs34173062 (p.Ser17Phe) in GWAS with AD-vulnerable brain features [46]. Furthermore, the latest large-scale GWAS meta-analysis based on data from IGAP (the International Genomics of Alzheimer Project) demonstrated significant genome-wide associations with AD for two missense variants of *SHARPIN* (rs34173062, p.Ser17Phe; and rs34674752, p.Pro294Ser) [47]. However, both rs34173062 and rs34674752 are extremely rare in East Asians, including Japanese population.

In conclusion, we identified a novel functional variant of *SHARPIN* that is significantly associated with an increased risk of LOAD in the Japanese population. Evidence that has accumulated since our first discovery of a LOAD-risk *SHARPIN* variant supports *SHARPIN* as an important LOAD-related gene. Elucidating the mechanism underlying the onset of LOAD requires further investigation into the physiologic roles of SHARPIN. LOAD is burdensome, not only for patients but also for their families and caregivers. Prevention and treatment of LOAD are urgent medical issues in developed countries such as Japan, which is rapidly becoming a super-aged society. Further investigation into the physiologic role of SHARPIN likely will clarify the mechanism of LOAD onset and will advance the quest for novel drug targets and innovative pharmaceutical approaches.

## Supplementary information

Supplemental Table 1

Supplemental Table 2

Supplemental Table 3

Supplemental Figure 1

## Data Availability

The datasets used or analyzed during the current study are available from the corresponding author on reasonable request.
