# Supplementary figures and images for: A functional variant of SHARPIN confers increased risk of late-onset Alzheimer’s disease
Source: J Hum Genet. 2021 Nov 5;67(4):203–8. doi: 10.1038/s10038-021-00987-x (PMC8948087; doi:10.1038/s10038-021-00987-x)

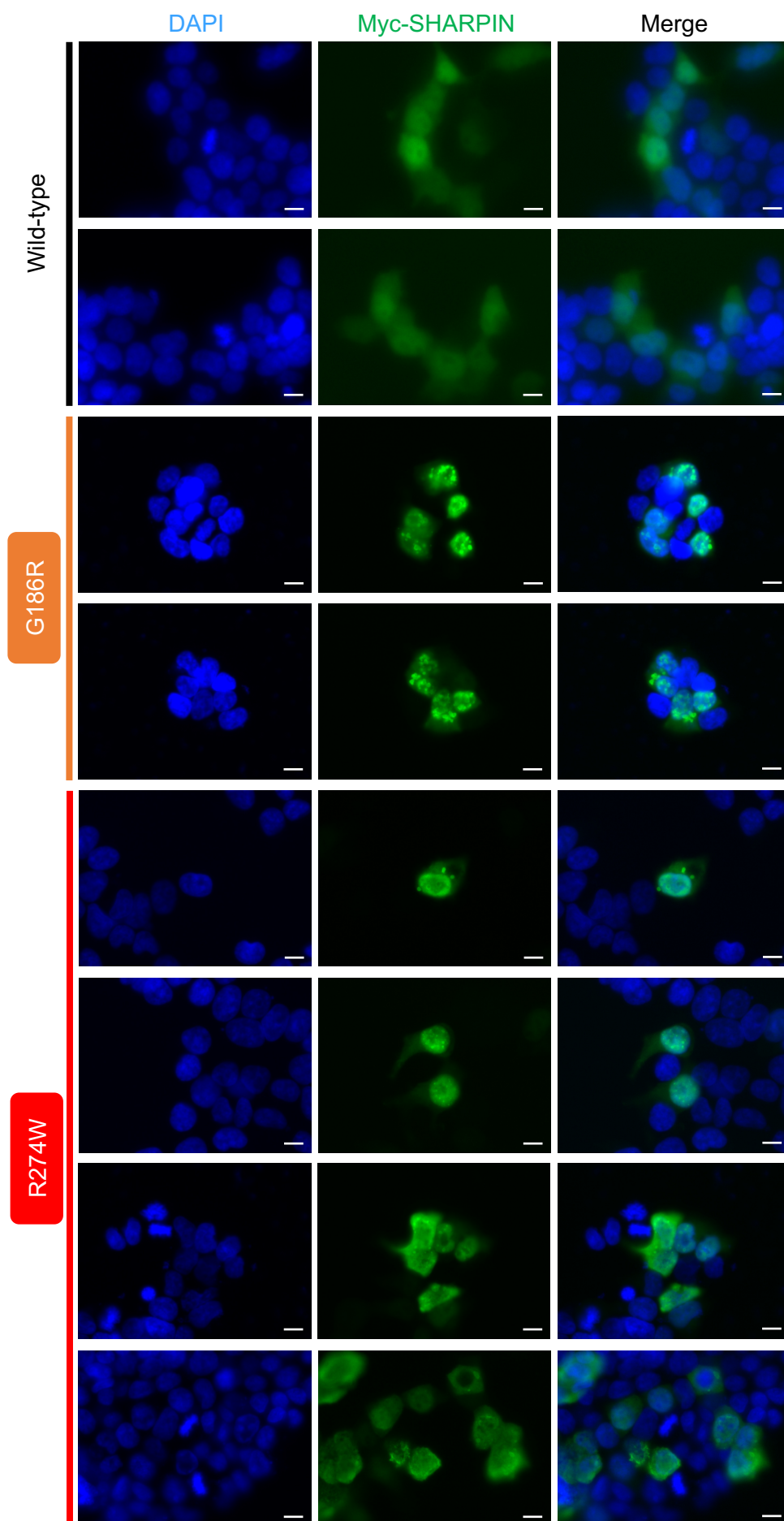

**Figure S1.** Detailed images of immunocytochemistry in Figure 2c, 2d, and 2e. Scale bar: 10  $\mu$ m.

Supplement: Supplementary file 4 — Supplemental Figure 1 [file 10038_2021_987_MOESM4_ESM.pdf]
